# The Depleting and Buffering Effects of Telecommuting on Wellbeing: Evidence From China During COVID-19

**DOI:** 10.3389/fpsyg.2022.898405

**Published:** 2022-05-11

**Authors:** Jinkai Cheng, Chao Zhang

**Affiliations:** ^1^School of Labor and Human Resources, Renmin University of China, Beijing, China; ^2^School of Economics, Zhejiang University, Hangzhou, China

**Keywords:** telecommuting, psychological detachment from work, wellbeing, job satisfaction, emotional exhaustion, family interfering with work, family–work enrichment

## Abstract

Meta-analytical research has demonstrated the benefits brought by telecommuting to wellbeing. However, we argue that such a setup in the course of the coronavirus disease pandemic exerts negative effects. On the basis of conservation of resources theory, this study determined how telecommuting depletes wellbeing (defined by job satisfaction and emotional exhaustion) through obstructing psychological detachment from work. Moreover, we incorporated family interfering with work and family–work enrichment as moderators that can buffer the negative effect of telecommuting on psychological detachment from work. Time-lagged field research was conducted with 350 Chinese employees, and findings largely supported our theoretical hypotheses. The elevated level of telecommuting results in minimal psychological detachment from work, which then leads to low wellbeing. Meanwhile, the negative effect of the extent of telecommuting on psychological detachment from work is reduced by family interfering with work. These findings extend the literature on telecommuting and psychological detachment from work through revealing why teleworkers present negative feelings during the pandemic.

## Introduction

The coronavirus disease (COVID-19) pandemic has remarkably increased remote work, whose implications for employees and organizations thus require thorough understanding (Malankowski and Wrycza, 2020; Leroy et al., 2021; Shockley et al., 2021; Zhang et al., 2021). In China, over 300 million workers relatively experienced telecommuting in March 2020. Meanwhile, the United States and Europe also experienced a multifold increase in remote work during the COVID-19 pandemic (Chong et al., 2020; Zhang et al., 2021). According to Allen et al. (2015), telecommuting refers to “*a work practice that involves members of an organization substituting a portion of their typical work hours (ranging from a few hours per week to nearly full-time) to work away from a central workplace—typically principally from home—using technology to interact with others as needed to conduct work tasks*.” The core argument behind telecommuting is that the boundaries between work and home became blurred for many, and employees are confronted with the need to simultaneously fulfill both work and family roles (Shockley et al., 2021).

Allen et al. (2015) reviewed the telecommuting literature and found that telecommuting is generally perceived as “good” for employees. In fact, meta-analytic evidence associates telecommuting with numerous indicators of enhanced wellbeing, including increased job satisfaction and decreased role stress (Gajendran and Harrison, 2007). On the contrary, other studies have revealed the negative implications of telecommuting for wellbeing (e.g., Cooper and Kurland, 2002; Golden and Veiga, 2005; Van der Elst et al., 2017; Wöhrmann and Ebner, 2021). For instance, Van der Elst et al. (2017) demonstrated that the extent of telecommuting is negatively related to wellbeing *via* the lack of social support from colleagues. Therefore, previous findings on the effect of telecommuting on wellbeing are rather inconsistent; the impact of telecommuting in particular contexts should be further explored (Allen et al., 2015). The COVID-19 pandemic has required several employees to work remotely, but evidence indicates that such employees may face particular constraints (Zhang et al., 2021), implying the significance of understanding the negative consequence of telecommuting. From a recent survey, employees under remote work during COVID-19 showed a decline in attitude after a work week (Zhang et al., 2021). However, few telecommuting studies have identified how telecommuting depletes the wellbeing of employees during the COVID-19 pandemic (Wöhrmann and Ebner, 2021). Furthermore, prior telecommuting studies have failed to investigate how to buffer against the depletion process. The limited research on the negative effect of telecommuting constrains theory and practice.

**Figure 1:**
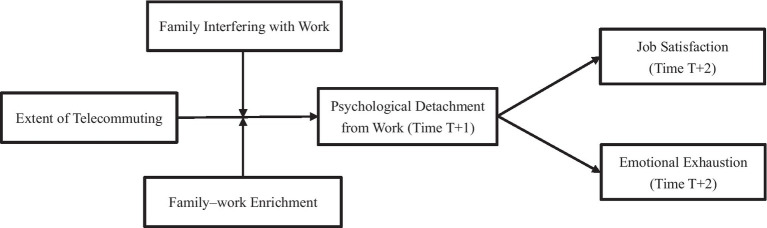
Hypothesized model (For simplicity, control variables are not included in this figure).

Our study aims to fill these gaps in our knowledge of telecommuting’s impact on wellbeing by using two widely accepted constructs of job satisfaction and emotional exhaustion (Gajendran and Harrison, 2007; Allen et al., 2015). First, on the basis of conservation of resources (COR) theory (Hobfoll, 1989), we construct a model linking telecommuting with changes in the wellbeing of employees, contending that telecommuting declines the wellbeing of employees because they hardly feel psychological detachment from work during their free time. As a result, the literature on telecommuting is expanded, considering that psychological detachment from work is regarded as a key factor to the wellbeing-depleting process of telecommuters. Second, we offer a more refined exposition of COR theory, stressing that resources must be invested by employees to recover from losses (Hobfoll et al., 2018). In doing so, the mechanisms of resource investment with regard to family aspects, such as family interfering with work (Delanoeije et al., 2019) and family–work enrichment (Heskiau and McCarthy, 2021), are regarded as the buffering mechanisms in our theoretical model. Hence, the predictors of psychological detachment from work from prior research, which concentrated only on job stressors or demands, are broadened (Sonnentag and Fritz, 2007; Sonnentag, 2018). Lastly, the theory is substantiated through analysis of the connection between psychological detachment from work and wellbeing in the aspect of telecommuting. This analysis proves that as a vital recovery experience, psychological detachment from work promotes the wellbeing of telecommuters. Figure 1 depicts our hypothesized model.

## Theory and Hypotheses

### Extent of Telecommuting and Psychological Detachment

Scholars classify employees into telecommuters and non-telecommuters by using a binary “yes or no” variable in the conceptualization and measurement of telecommuting, but they also indicate that research should consider the telecommuting degree of an individual (Allen et al., 2015; Golden and Eddleston, 2020). The binary variable disregards the variation in the way telecommuters work remotely and the differences among telecommuters themselves (Golden et al., 2008; Golden and Eddleston, 2020). The telecommuting experiences of individuals during the COVID-19 pandemic vary between occasional telecommuting and regular telecommuting (i.e., multiple days per week; Golden and Veiga, 2005); thus, the influences on their wellbeing differ. In accordance with earlier literature on telecommuting (Golden and Veiga, 2005; Golden et al., 2006, 2008; Allen et al., 2015; Golden and Gajendran, 2019; Golden and Eddleston, 2020), the extent of telecommuting and its effects on employees are focused on in the current study.

On the basis of COR theory (Hobfoll, 1989), the extent of telecommuting is predicted to influence psychological detachment from work negatively. The theory indicates that individuals aim at protecting their present resources, obtaining new ones, and inhibiting their loss (Hobfoll, 1989). The conservation and cultivation of resources lead to positive wellbeing (Gilbert et al., 2018), but their loss results in psychological distress, anxiety, and depression (Kessler et al., 1988; Halbesleben et al., 2014). During the transition to remote work, workers may lose resources at work, such as coworker support, and in life, such as social isolation and services, simultaneously (Wanberg et al., 2020).

Accordingly, the boundaries between work and home are blurred by telecommuting, thus, the teleworking days of employees are considerably affected by work-to-home transitions (namely, work disruptions to address home issues during work time; Delanoeije et al., 2019). This condition causes job and challenge stressors, including excessive workload and time pressure. Consequently, they likely handle exhausting circumstances by engaging considerably in home-to-work transitions (namely, home disruptions to address work issues after work time; Delanoeije et al., 2019), doing minimal physical exercise, or decreasing sleeping hours (Sonnentag, 2018). Large-scale cohort research has verified the lack of physical activity and quality sleep among individuals having jobs with massive workload and high strain (Nixon et al., 2011; Stults-Kolehmainen et al., 2014; Oshio et al., 2016). Their recovery experience is likely influenced by the decrease in recovery activity and process (Sonnentag, 2018). Sonnentag (2018) and Sonnentag et al. (2008) recommended that a significant recovery experience is psychological detachment from work, which indicates “*an individual’s sense of being away from the work situation*” (Etzion et al., 1998) *and implies not only refraining from performing job-related tasks, but also mentally disconnecting from the job during nonwork time* (Sonnentag et al., 2008). Excessive work demands, including time pressure, decrease psychological detachment in the long run (Kinnunen and Feldt, 2013). Therefore, we can assume that telecommuting leads to difficulty in psychological detachment from work.

*Hypothesis 1*: The extent of telecommuting will be negatively related to psychological detachment from work.

### Mediating Role of Psychological Detachment

The negative relation between telecommuting and wellbeing has been tackled in some research (e.g., Golden and Veiga, 2005; Van der Elst et al., 2017; Wöhrmann and Ebner, 2021), but they did not pay much attention to the viable mechanisms for such relation. For instance, Golden and Veiga (2005) indicated that the extent of telecommuting and job satisfaction have a curvilinear connection and that job satisfaction stabilizes as the extent of telecommuting intensifies. However, the mediating mechanisms between them are not clear; as suggested by Golden and Veiga (2005), future research could further unravel the complexities inherent in this work. Therefore, the factors behind the poor wellbeing of telecommuters must be examined to fully comprehend the connection between telecommuting and wellbeing.

With reference to the studies of Lennard et al. (2019), Chawla et al. (2020), and Zhong et al. (2021) emotional exhaustion (i.e., personal ill-being) and job satisfaction (i.e., work-related wellbeing), which are the most considered wellbeing types, are evaluated in the current study. These types are the basic indices of wellbeing used in theoretical models of telecommuting in considerable research (e.g., Golden and Veiga, 2005; Golden, 2006a,b; Gajendran and Harrison, 2007; Sardeshmukh et al., 2012).

Based on empirical studies, good wellbeing is realized with psychological detachment from work after working hours (Hülsheger et al., 2014; Wendsche and Lohmann-Haislah, 2017; Bennett et al., 2018; Sonnentag, 2018). A meta-analysis has also demonstrated that detachment from work and self-rated mental state are positively correlated, leading to decreased exhaustion, enhanced life satisfaction, and improved wellbeing (Wendsche and Lohmann-Haislah, 2017). Detachment from work is an effective recovery approach as it helps in replenishing resources through mentally separating employees from work (Wendsche and Lohmann-Haislah, 2017). From the perspective of telecommuting, psychological detachment from work is assumed to decrease the degree of emotional exhaustion and increase the extent of job satisfaction through reducing work-related stress after working and restoring lost resources while working (Sonnentag and Fritz, 2007; Siltaloppi et al., 2011). According to a recent study, as a valuable recovery experience, psychological detachment from work favorably influences the wellbeing of employees for the following workday (Chawla et al., 2020).

On the basis of Hypothesis 1, which indicates that the extent of telecommuting exerts a negative effect on psychological detachment from work, we assume that psychological detachment from work mediates the relationship between the extent of telecommuting and the wellbeing of employees (i.e., emotional exhaustion and job satisfaction).

*Hypothesis 2*: The extent of telecommuting presents indirect effects on (a) emotional exhaustion and (b) job satisfaction *via* psychological detachment from work.

### Moderating Effect of Family Interfering With Work

In China, numerous employees were unexpectedly required to work from home, although they were not willing or were not given the opportunity to telecommute in the past (Chong et al., 2020; Zhang et al., 2021). For such employees who were forced to work remotely, the boundaries between work and family became hazy (Shockley et al., 2021). That is, they were considerably affected by family life through family interfering with work (Duxbury et al., 1992; Carlson et al., 2000) and family–work enrichment (Lin et al., 2020; Heskiau and McCarthy, 2021; Wu et al., 2021).

Family interfering with work is defined as “*interruptions of work activities to deal with family demands*” (Delanoeije et al., 2019), which can be regarded as a key to work–family conflict (Carlson et al., 2000). Golden et al. (2006) determined that extensive telecommuting leads to increased family–work conflict. The work time of telecommuters may be remarkably intervened by family activities. Family interfering with work is expected to buffer the negative effect of telecommuting on psychological detachment from work. While performing family activities, including cooking and supervising children, during work time, employees likely transit from playing work roles to playing family roles. Consequently, such employees likely detach mentally from work-related responsibilities after working hours. By contrast, telecommuters who are not required to address family needs may still be dominated by work rather than doing family activities even after work time. Psychological detachment from work may be difficult for them.

*Hypothesis 3*: Family interfering with work moderates the negative relationship between the extent of telecommuting and psychological detachment from work; thus, such a negative relationship weakens as the family interfering with work intensifies.

### Moderating Effect of Family–Work Enrichment

Family–work enrichment is viewed as “*the degree to which developmental, affective, social capital, and efficiency gains in family domain enhance employee conditions in the work domain*” (Lin et al., 2020). The negative effect of telecommuting on psychological detachment from work is expected to be buffered by family–work enrichment. COR theory states that lost resources can be restored by resources from other fields (Hobfoll, 2001). On this basis, constructive encounters with the family can be beneficial to the work of individuals, resulting in their efficiency at work (Lin et al., 2020); hence, telecommuters can accomplish their work responsibilities effectively and achieve psychological detachment from work after working hours. Family–work enrichment also promotes optimistic mood and job satisfaction (Carlson et al., 2011), thereby increasing resource recovery experience, such as psychological detachment from work. Thus, we establish the following hypothesis:

*Hypothesis 4*: Family–work enrichment moderates the negative relationship between the extent of telecommuting and psychological detachment from work; consequently, such a negative relationship declines when the family–work enrichment rises.

## Materials and Methods

### Contextual Background, Procedure, and Participants

We used snowball sampling approach (Lin et al., 2020) to recruit participants across different industries, occupations, organizations, and locations in China. The data collection process commenced in January 2021 and ended in April 2021. We asked 5 MBA students in a large university in China to help invite participants to take part in our study. MBA students also asked their friends to help further advertise the study and invite participants. Employees who were forced to telecommute as a result of COVID-19 were eligible for participating in our study. MBA students and their friends helped distribute surveys by sending electronic questionnaires to corresponding respondents.

Data were collected at three time points, with an interval of 1 month on average, to alleviate common method bias concerns. During Time 1 (T1), the weekly work and telework hours, family interfering with work, and family–work enrichment of each respondent were measured. Control variables, such as their gender, age, and educational achievement, were also determined. During Time 2 (T2), 1 month after T1, their psychological detachment from work, professional isolation, and employee trust were evaluated. During Time 3 (T3), 2 months after T1, their job satisfaction and emotional exhaustion were assessed. An exclusive telephone number was used to match the sample.

At T1, we gathered 765 data from the respondents. Among them, *N* = 167 (21.8%) were eliminated due to failure in quality checks or lack of variance. A sample of *N* = 598 (78.2%) remained and were requested for T2. At T2 (77.6%), 464 valid respondents were identified and invited for T3. At T3, 350 valid respondents were noted, indicating an effective response rate of 75.4%. Thus, a final sample of *N* = 350 was generated.

Among the participants, 67.7% were females, 83.4% had been working at their respective companies for more than 3 years. Their age was distributed as follows: 20–25 years: 3.7% (*N* = 13); 26–30 years: 20.6% (*N* = 72); 31–40 years: 50.9% (*N* = 178); 41–50 years: 20.3% (*N* = 71); 51–60 years: 4.6% (*N* = 16). On average, their weekly work hours were 45.22 (SD = 8.92) and telework hours were 7.76 (SD = 10.98). They came from diverse industries, such as manufacturing (28%), Internet or financial sector (19.1%), civil service (9.1%), education or culture (8.3%), and specialized fields of law or research (2.6%).

### Measures

The measures for this study were administered in Chinese. In accordance with Brislin’s (1970) procedure, the items were translated into Chinese by the first author and back-translated into English by the second author. The original and back-translated versions were thoroughly reviewed by the authors. To guarantee conceptual similarity, the translated version was amended when discrepancies were identified. Except as otherwise specified, a five-point Likert scale (1 = strongly disagree to 5 = strongly agree) was used for all items.

#### Extent of Telecommuting

The measure proposed by Golden and Veiga (2005) and adopted in numerous studies (e.g., Golden et al., 2006, 2008; Golden and Eddleston, 2020) was used to evaluate the extent of telecommuting. The regular work schedule and the hours spent on telecommuting during regular work schedule within a typical work week of the respondents were determined. The extent of telecommuting was measured by dividing telecommuting hours by regular work hours. Responses ranged from 0 to 100%, with a mean of 17%.

#### Psychological Detachment From Work

Sonnentag and Fritz (2007) developed a four-item scale for measuring psychological detachment from work, which was employed in the current study. Other studies (e.g., Hülsheger et al., 2014; Haun et al., 2018; Van Laethem et al., 2018) have also adopted this scale. Among the items is “In my free time after work, I forget about the work today.” The reliability was *α* = 0.921.

#### Job Satisfaction

A three-item scale established by Hackman et al. (1980) and applied in several studies (e.g., Froese et al., 2019) was used for measuring job satisfaction. Among the items is “I am fairly satisfied with my job.” The reliability was *α* = 0.926.

#### Emotional Exhaustion

Teuchmann et al. (1999) developed a two-item scale for measuring emotional exhaustion, which has been used in studies (e.g., Chong et al., 2020). This scale was applied in the current study. The items are “To what extent do you feel emotionally drained by work?” and “How much do you feel burned out from work?” Respondents may choose from 1 to 5, where 1 = almost never and 5 = almost always, as a response. The reliability was *α* = 0.84.

#### Family Interfering With Work

A four-item subscale, family interfering with work, from the work–family conflict scale by Carlson et al. (2000) was used to measure family interfering with work. Among the items is “Because I am often stressed from family responsibilities, I have a hard time concentrating on my work.” The reliability was *α* = 0.924.

#### Family–Work Enrichment

Family–work enrichment was assessed through a nine-item scale, which was proposed by Carlson et al. (2006) and has been employed by studies (e.g., Wu et al., 2021). The scale comprises three factors, namely, *family to work development* (3 items), *family to work affect* (3 items), and *family to work efficiency* (3 items). The items include “My involvement in my family increases my knowledge and this helps me be a better worker (family to work development),” “My involvement in my family puts me in a good mood and this helps me be a better worker (family to work affect),” and “My involvement in my family requires me to avoid wasting time at work and this helps me be a better worker (family to work efficiency).” The reliability for the three items was *α* = 0.936, 0.915, and 0.826, respectively.

#### Control Variables

The demographic characteristics of teleworkers, such as *gender* (male = 1, female = 2), *age*, and *education*, and work-related variables, namely, *job tenure*, *job type*, *company type*, and *hours worked*, were regarded as control variables, considering that they reflect individual and work differences, which could influence the wellbeing of teleworkers (Allen et al., 2015; Golden and Eddleston, 2020; Trougakos et al., 2020).

To ensure that the results are robust to the inclusion of potential confounders, we controlled additional variables, including *professional isolation* and *employee trust*, which have been proven critical mediators between telecommuting and outcomes (Cooper and Kurland, 2002; Golden et al., 2008). Professional isolation refers to “*a state of mind or belief that one is out of touch with others in the workplace*” (Golden et al., 2008). The level of professional isolation was determined using a seven-item measure introduced by Golden et al. (2008). A sample item is “I feel left out on activities and meetings that could enhance my career during telecommuting.” The reliability was *α* = 0.92. Meanwhile, employee trust was measured *via* a three-item scale proposed by Brower et al. (2000). Among the items is “I trust the organization I work for.” The reliability was *α* = 0.859.

## Results

Mplus Version 8.3 was utilized for data analysis. Before the analysis, the measurement model was assessed through confirmatory factor analysis. The model comprised seven factors, namely, psychological detachment from work, job satisfaction, emotional exhaustion, family interfering with work, and the three factors of family–work enrichment. Table 1 verifies the acceptable fit (*χ^2^* = 388.623, *df* = 188, RMSEA = 0.055, CFI = 0.97, TLI = 0.963, SRMR = 0.038, *p* < 0.01) and better fit of the full measurement model in comparison with a one-factor model (*χ^2^* = 4195.67, *df* = 209, RMSEA = 0.233, CFI = 0.395, TLI = 0.331, SRMR = 0.180, *p* < 0.01) or any alternative models when any pair of the multilevel variables was loaded on one factor. Therefore, our results were insignificantly affected by common method variance. The descriptive statistics and correlations of study variables are shown in Table 2.

**Table 1:** Confirmatory factor analysis.

Factor model	# of factors	*χ^2^*	*df*	*χ^2^/df*	RMSEA	CFI	TLI	SRMR
Full measurement model	7	388.623	188	2.067	0.055	0.97	0.963	0.038
All family–work enrichment collapsed	5	988.787	199	4.969	0.106	0.88	0.861	0.047
Job satisfaction and emotional exhaustion collapsed^*^	6	586.481	194	3.023	0.076	0.94	0.929	0.049
Job satisfaction and family–work enrichment collapsed^*^	4	1687.422	203	8.312	0.145	0.775	0.744	0.084
Job satisfaction and psychological detachment collapsed^*^	6	1518.335	194	7.826	0.140	0.799	0.761	0.117
All collapsed to one factor	1	4195.67	209	16.14	0.233	0.395	0.331	0.180

*N = 350*.

* *Variables were selected in consideration of their relatively high correlations*.

**Table 2:** Means, standard deviations, and correlations of study variables.

Variables	Mean	SD	1	2	3	4	5	6	7	8	9	10	11	12	13	14
1. Gender (T1)	1.68	0.47	1													
2. Age (T1)	3.01	0.86	−0.13^*^													
3. Education (T1)	2.28	1.13	0.1	0.21^**^												
4. Job tenure (T1)	4.28	1.67	−0.07	0.49^**^	0.01											
5. Job type (T1)	2.44	1.63	−0.05	0.17^**^	0.27^**^	0.12^*^										
6. Company type (T1)	4.2	1.8	−0.02	0.16^**^	0.06	0.13^*^	0.24^**^									
7. Hours worked (T1)	45.22	8.92	−0.15^**^	0.11^*^	0.09	0.02	0.11^*^	−0.01								
8. Professional isolation (T2)	2.63	0.91	−0.01	−0.04	0.01	−0.001	0.04	0.001	−0.04							
9. Employee trust (T2)	4.05	0.7	−0.04	0.21^**^	0.14^*^	0.17^**^	0.23^**^	0.1	0.03	−0.08						
10. Extent of telecommuting (T1)	0.17	0.23	−0.05	−0.01	−0.01	−0.06	−0.07	−0.1	−0.004	−0.001	−0.03					
11. Family interfering with work (T1)	2.36	0.94	−0.004	−0.14^*^	−0.13^*^	−0.05	−0.01	−0.02	0.02	0.06	−0.14^**^	0.002				
12. Family–work enrichment (T1)	3.92	0.73	0.06	0.19^**^	0.14^*^	0.17^**^	0.22^**^	0.14^*^	−0.02	−0.04	0.51^**^	−0.11^*^	−0.12^*^			
13. Psychological detachment (T2)	3.11	1.02	0.03	−0.08	−0.01	−0.04	0.04	−0.01	−0.03	−0.07	0.05	−0.15^**^	0.09	0.06		
14. Job satisfaction (T3)	3.92	0.73	0.01	0.25^**^	0.15^**^	0.22^**^	0.17^**^	0.15^**^	0.08	−0.03	0.75^**^	−0.02	−0.22^**^	0.52^**^	0.15^**^	
15. Emotional exhaustion (T3)	2.68	1.06	0.05	−0.23^**^	−0.07	−0.13^*^	−0.1	−0.11^*^	−0.02	−0.22^**^	−0.48^**^	−0.02	0.18^**^	−0.31^**^	−0.11^*^	−0.49^**^

*N = 350. SD = Standard deviation; Gender is coded 1 = male, 2 = female*. *T1 = Data collected at Time 1; T2 = Data collected at Time 2; T3 = Data collected at Time 3*.

* *p ≤ 0.05;*

** *p ≤ 0.01 (two-tailed)*.

The hypotheses were tested through multiple regression analyses, and the results are indicated in Table 3. Hypothesis 1 stated that the extent of telecommuting negatively influences psychological detachment from work. A model with psychological detachment from work regressed on only control variables was estimated first (Model 1). To test Hypothesis 1, the extent of telecommuting was incorporated into the previous model. The results of this model (Model 2) demonstrated the statistical significance of the coefficient for the effect (*b* = −0.639, *p* ≤ 0.01). Empirical verification for Hypothesis 1 was therefore achieved.

**Table 3:** Hierarchical regression results.

Variable	Psychological detachment					Job satisfaction			Emotional exhaustion		
	Model 1	Model 2	Model 3	Model 4	Model 5	Model 6	Model 7	Model 8	Model 9	Model 10	Model 11
Intercept	3.335^**^	3.199^**^	3.114^**^	3.525^**^	3.532^**^	0.027	0.015	−0.312	5.201^**^	5.253^**^	5.584^**^
Gender	0.045	0.026	0.038	0.021	0.021	0.081	0.082	0.079	0.04	0.035	0.038
Age	−0.102	−0.083	−0.079	−0.098	−0.098	0.043	0.043	0.051	−0.172^*^	−0.171^*^	−0.18^**^
Education	−0.01	0.001	0.006	−0.01	−0.01	0.022	0.022	0.022	0.011	0.011	0.011
Job tenure	−0.005	−0.011	−0.008	−0.011	−0.011	0.032	0.032	0.033	0.009	0.008	0.007
Job type	0.032	0.024	0.026	0.026	0.026	−0.017	−0.017	−0.019	0.013	0.012	0.014
Company type	−0.008	−0.015	−0.013	−0.016	−0.016	0.028	0.029^*^	0.03^*^	−0.032	−0.034	−0.036
Hours worked	−0.003	−0.003	−0.001	−0.003	−0.003	0.004	0.004	0.005	0.002	0.002	0.002
Professional isolation	−0.078	−0.082	−0.081	−0.077	−0.077	0.027	0.027	0.034	0.217^**^	0.217^**^	0.21^**^
Employee trust	0.083	0.099	0.107	0.061	0.06	0.764^**^	0.764^**^	0.756^**^	−0.667^**^	−0.667^**^	−0.66^**^
TELE		−0.639^**^	−0.692^**^	−0.627^**^	−0.632^**^		0.04	0.099		−0.17	−0.23
Family interfering with work		0.106	0.101								
Family–work enrichment				0.042	0.043						
TELE × Family interfering with work			−0.773^**^								
TELE × Family–work enrichment					−0.025						
Psychological detachment								0.093^**^			−0.094^*^
*R^2^*	0.018	0.047^*^	0.071^**^	0.039	0.039	0.591^**^	0.591^**^	0.607^**^	0.288^**^	0.29^**^	0.298^**^

*N = 350. TELE = Extent of telecommuting. TELE, family interfering with work, and family–work enrichment were centered before the interaction. Unstandardized regression coefficients are reported*.

* *p ≤ 0.05;*

** *p ≤ 0.01 (two-tailed)*.

In Hypothesis 2a, psychological detachment from work was proposed to mediate the positive relationship between the extent of telecommuting and emotional exhaustion. From Table 3, the extent of telecommuting and psychological detachment from work were negatively correlated (Model 2, *b* = −0.639, *p* ≤ 0.01). Likewise, psychological detachment from work and emotional exhaustion showed a negative correlation (Model 11, *b* = −0.094, *p* ≤ 0.05). Moreover, no zero was included in the 95% confidence intervals of the indirect consequence of the extent of telecommuting on emotional exhaustion *via* psychological detachment from work (estimate = 0.086, 95% CI [0.0049, 0.2097]). Hypothesis 2a thus received empirical support. In Hypothesis 2b, psychological detachment from work was suggested to mediate the negative relationship between the extent of telecommuting and job satisfaction. Table 3 presents that telecommuting degree and psychological detachment from work were negatively correlated (Model 2, *b* = −0.639, *p* ≤ 0.01), whereas psychological detachment from work and job satisfaction were positively correlated (Model 8, *b* = 0.039, *p* ≤ 0.01). Similar to the result for Hypothesis 2a, no zero was included in the 95% confidence intervals of the indirect consequence of the extent of telecommuting on job satisfaction *via* psychological detachment from work (estimate = −0.077, 95% CI [−0.1775, −0.0107]). Therefore, Hypothesis 2b gained empirical support.

**Figure 2:**
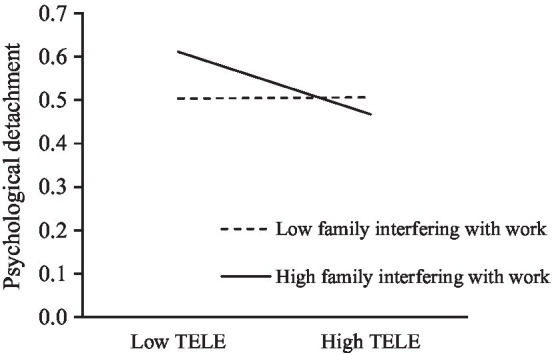
Moderation effects of family interfering with work.

In Hypothesis 3, we argued that the connection between the extent of telecommuting and psychological detachment from work at high levels of family interfering with work is less negative than that at low levels. Table 3 demonstrates that the coefficient for the interaction effect became negative and significant (*b* = −0.773, *p* ≤ 0.01) after the interaction term of the extent of telecommuting and family interfering with work was added to Model 3. This result indicated the significant negative effect of family interfering with work on the scale of telecommuting–psychological detachment. Then, we plotted the interaction between telecommuting and family interfering with work in Figure 2 in accordance with two conditional values: the standard deviation above and below the mean. On the basis of simple slope analyses, the negative relation between telecommuting and psychological detachment was lower (simple slope = −0.357, SE = 0.09, *p* ≤ 0.01) when family interfering with work was high (+1 SD) than when family interfering with work was low (−1 SD; simple slop = 0.007, SE = 0.081, *n.s.*), thereby supporting Hypothesis 3.

Hypothesis 4 postulated that family–work enrichment moderates the association between the extent of telecommuting and psychological detachment from work. As presented in Table 3, the coefficient for the interaction effect was statistically insignificant (*b* = −0.025, *n.s.*) after the interaction term between the extent of telecommuting and family–work enrichment was integrated into Model 5. Consequently, Hypothesis 4 was unsupported.

## Discussion

Our objective in this study was to clarify how and when telecommuting decreases, the wellbeing of employees on the basis of COR theory. Results from a sample of employees in China during COVID-19 provided strong support for our research model. One finding was that extensive telecommuting and declined wellbeing (*via* increased emotional exhaustion and decreased job satisfaction) through reduced psychological detachment from work were indirectly related. Moreover, telecommuting and psychological detachment from work exhibited a weaker correlation given higher family interfering with work. These findings contribute to telecommuting research and practices in the time of COVID-19 pandemic.

### Theoretical Implications

First, our study adds to the telecommuting literature through identifying psychological detachment from work as a key mediator of the relationship between the extent of telecommuting and the wellbeing of employees. Specifically, our study provides evidence on why telecommuting depletes the wellbeing of employees. Prior research on telecommuting typically focused on boundaryless working hours, relationship with coworkers, and social support from colleagues (Van der Elst et al., 2017; Wöhrmann and Ebner, 2021) as mediators of the negative relationship between the extent of telecommuting and the wellbeing of employees. Less attention, however, has been paid to the deprivation of recovery-enhancing process as a potential factor of lost wellbeing of telecommuters. Consistent with the recent theory that resource loss has a spiraling nature because resource loss is more powerful than resource gain (Hobfoll et al., 2018), we draw insights from COR theory to explain how telecommuting can deprive recovery process, such as psychological detachment from work during non-work time, which in turn creates resource loss spiral whereby the lost wellbeing gain in both impact and momentum. Our results demonstrate that telecommuting likely leads to negative implications for wellbeing because of the loss of psychological detachment from work during non-work time. This finding is in line with the suggestion of Sonnentag (2018) that psychological detachment from work, as an important recovery experience, is a key indicator of good wellbeing.

Second, prior research on telecommuting typically concentrated on how job factors (e.g., task interdependence and job discretion; Golden and Veiga, 2005) and organizational factors (e.g., perceived organizational telework task support and group belongingness; Chong et al., 2020; Bennett et al., 2021) influence the relations between telecommuting and outcomes. However, less attention has been paid to family factors. As mentioned by Shockley et al. (2021), forced remote workers were confronted with the immediate and ongoing need to simultaneously fulfill both work and family roles. Therefore, on the basis of COR theory, we contribute to broadening the understanding of how the depleting effects of telecommuting on wellbeing can be relieved by family through showing the moderating effects of family interfering with work and family–work enrichment. Our findings demonstrate that psychological detachment from work is most likely to develop when complementarity is created by the match of low extent of telecommuting with high family interfering with work. This finding is in line with the self-expansion approach (Mattingly and Lewandowski, 2013), which indicates that new activities (in this case, telecommuting) support the development of people’s resources through their engagement with the new activities. This theory can explain an intraindividual expansion driven by an external condition, such as working from home (Toscano and Zappalà, 2021). Although not initially postulated by this study, according to this theory, the moderating effect of family interfering with work on the relationship between the extent of telecommuting and psychological detachment from work can be explained by the energizing effect and mutual exchange of resources that occur between close people, leading to self-expansion. In the case of employees who work from home, interference from family, such as attending to the needs of their children, and engaging in arguments with their spouse, may motivate the employees to seek self-expansion and become considerably active to fulfill both work and family roles (Toscano and Zappalà, 2021). This self-expansion may lead employees to increased fulfillment in their work and result even in enhanced psychological detachment from work during non-work time.

However, the moderating effect of family–work enrichment on the connection between telecommuting degree and psychological detachment from work hypothesized in this study is not supported. Lin et al. (2020) found that employees who place importance on their family role regard positive family events as beneficial. Accordingly, we assume that the moderating role of family–work enrichment may be influenced by the preferences of employees. An individual with a home protection preference may considerably benefit from family–work enrichment and likely develop psychological detachment from work. On the contrary, an individual with a work protection preference may view family–work enrichment as minimally beneficial to psychological detachment from work. This issue requires further exploration.

Third, we contribute to the literature on psychological detachment from work by substantiating that telecommuting degree and psychological detachment from work are negatively correlated. Previous research on the antecedents of psychological detachment from work focused on work characteristics, including job demands and heavy work investment (Sonnentag and Fritz, 2007; Wendsche and Lohmann-Haislah, 2017; Sonnentag, 2018). On the contrary, we combine telecommuting and family factors to demonstrate the difficulty faced by telecommuters with regard to psychological detachment from work. We illustrate the negative effect of telecommuting on psychological detachment from work, as well as the buffering effects of family interfering with work and family–work enrichment. In contrast to the notion that psychological detachment from work is solely determined by work factors (Sonnentag et al., 2012; Sonnentag and Fritz, 2015), our research indicates that it is a vital recovery experience providing an interactive outcome between work and family.

Lastly, the positive connection between psychological detachment from work and self-rated wellbeing (i.e., decreased emotional exhaustion and increased job satisfaction) in the telecommuting context demonstrated in this study adds to the (psychological) detachment literature. This finding aligns with that of previous research (e.g., Wendsche and Lohmann-Haislah, 2017; Sonnentag, 2018), which exhibited that detachment positively influences the wellbeing of employees in non-telecommuting contexts. Nonetheless, to our best knowledge, such a relation has not been tackled extensively and not been tested empirically in the telecommuting context. Through illustrating certain significant effects within the context of telecommuting, we present the first evidence that not only the employees who work in an office environment are affected but also the employees who work from home.

### Practical Implications

Our research provides valuable practical implications. First, our study advises that telecommuters and their managers should exercise caution when telecommuting extensively, particularly when telecommuters were previously unwilling or never offered the opportunity to telecommute, because this arrangement may potentially decline their wellbeing in the course of the COVID-19 pandemic. Second, managers should encourage telecommuters to take measures to help themselves apply psychological detachment from work after working hours; such measures include improvements in physical exercise, sleep quality, and sleep quantity, which have been regarded as effective recovery activities (Sonnentag, 2018). Lastly, family interfering with work remarkably benefits recovery when facing a high level of telecommuting. Thus, we encourage telecommuters to take the initiative to master the rhythm of work and have a rest after working for a while instead of focusing on thinking and working for long periods of time to preserve energy and prevent exhaustion.

### Limitations and Future Research Directions

The findings from this study must be applied in consideration of the following limitations. First, with respect to methodology, all the study variables were collected from the same source. Though we incorporated three measurement points into our design, this does raise possible concerns about common method variance. Thus, we recommend future research to explore our findings experimentally or use other kinds of measurements, such as objective measures (e.g., extent of telecommuting based on official records). Second, given the limited perspective of COR theory, future research could make further developments on the basis of the understanding of the depleting and buffering effects of telecommuting on wellbeing. For example, the environmental circumstances and personality differences of employees might be investigated, considering that these aspects influence their nature and degree of resource depletion (Golden, 2006a). Third, only employees from Chinese companies were included in our study sample. Consequently, the findings may not apply to employees in other countries. Thus, future research should examine the theoretical model by using samples from western countries or individualistic societies. Moreover, given that the average of telecommuting hours (7.76) in this study is low, the generalizability of our findings may also be limited. Future research should replicate our findings using the samples of extensive versus occasional telecommuting to ensure the applicability of our results.

## Conclusion

Interests in telecommuting, as a presumed concept, have dramatically increased in the recent years, especially amid the COVID-19 pandemic (Cooke et al., 2020; Malankowski and Wrycza, 2020; Zhang et al., 2021). Telecommuting models have deviated from the predictors of the performance and wellbeing of remote workers (e.g., Shockley et al., 2021). Our study aims to add to and not negate previous research. Gajendran and Harrison (2007) proposed the concept of “telecommuting paradox”; that is, telecommuting results in mutually incompatible consequences for employees. If telecommuting increases autonomy and decreases work–family conflict, then it may lead to enhanced job-related attitudes, improved performance, and reduced stress. However, while telecommuting, work relationships might be affected, and career advancement might be obstructed. Therefore, the effect of telecommuting must be explored within a specific context. In this study, we stress that employees who were forced to telecommute as a result of COVID-19 likely experienced negative feelings. This study aims not only to challenge previous views but also to encourage future research to explore other theories on telecommuting.

## Data Availability Statement

The raw data supporting the conclusions of this article will be made available by the authors, without undue reservation.

## Ethics Statement

Ethical review and approval was not required for the study on human participants in accordance with the local legislation and institutional requirements. The patients/participants provided their written informed consent to participate in this study.

## Author Contributions

JC designed the main idea, collected and analysed the data. CZ wrote the manuscript and modified the research design. All authors contributed to the article and approved the submitted version.

## Conflict of Interest

The authors declare that the research was conducted in the absence of any commercial or financial relationships that could be construed as a potential conflict of interest.

## Publisher’s Note

All claims expressed in this article are solely those of the authors and do not necessarily represent those of their affiliated organizations, or those of the publisher, the editors and the reviewers. Any product that may be evaluated in this article, or claim that may be made by its manufacturer, is not guaranteed or endorsed by the publisher.
